# Effect of isolated left bundle-branch block on biventricular volumes and ejection fraction: a cardiovascular magnetic resonance assessment

**DOI:** 10.1186/s12968-018-0457-8

**Published:** 2018-09-20

**Authors:** Shadi Akhtari, Michael L. Chuang, Carol J. Salton, Sophie Berg, Kraig V. Kissinger, Beth Goddu, Christopher J. O’Donnell, Warren J. Manning

**Affiliations:** 1 Department of Medicine, Cardiovascular Division, Beth Israel Deaconess Medical Center, Harvard Medical School, 330 Brookline Avenue, Boston, MA 02215 USA; 2 The NHLBI’s Framingham Heart Study, Framingham, MA USA; 3 Department of Radiology, Beth Israel Deaconess Medical Center, Harvard Medical School, Boston, MA USA; 4 Cardiology Section, Veterans Affairs Healthcare System, Boston, MA USA

**Keywords:** Left bundle branch block, Cardiovascular magnetic resonance, Left ventricular ejection fraction, Left ventricular size, Cardiac function

## Abstract

**Background:**

Left bundle branch block (LBBB) is associated with abnormal left ventricular (LV) contraction, and is frequently associated with co-morbid cardiovascular disease, but the effect of an isolated (i.e. in the absence of cardiovascular dissease) LBBB on biventricular volumes and ejection fraction (EF) is not well characterized. The objective of this study was to compare LV and right ventricular (RV) volumes and EF in adults with an isolated LBBB to matched healthy controls and to population-derived normative values, using cardiovascular magnetic resonance (CMR) imaging.

**Methods:**

We reviewed our clinical echocardiography database and the Framingham Heart Study Offspring cohort CMR database to identify adults with an isolated LBBB. Age-, sex-, hypertension-status, and body-surface area (BSA)-matched controls were identified from the Offspring cohort. All study subjects were scanned using the same CMR hardware and imaging sequence. Isolated-LBBB cases were compared with matched controls using Wilcoxon paired signed-rank test, and to normative reference values via Z-score.

**Results:**

Isolated-LBBB subjects (*n* = 18, 10F) ranged in age from 37 to 82 years. An isolated LBBB was associated with larger LV end-diastolic and end-systolic volumes (both *p* < 0.01) and lower LVEF (56+/− 7% vs. 68+/− 6%; *p*  <0.001) with similar myocardial contraction fraction. LVEF in isolated LBBB was nearly two standard deviations (Z = − 1.95) below mean sex and age-matched group values. LV stroke volume, cardiac output, and mass, and all RV parameters were similar (p = NS) between the groups.

**Conclusions:**

Adults with an isolated LBBB have greater LV volumes and markedly reduced LVEF, despite the absence of overt cardiovascular disease. These data may be useful toward the clinical interpretation of imaging studies performed on patients with an isolated LBBB.

## Background

The prevalence of left bundle-branch block (LBBB) in the general population has been estimated to range from 0.2 to 1.1% [1–3]. LBBB is often associated with underlying cardiovascular abnormalities such as coronary artery disease, hypertension, or dilated cardiomyopathy [4, 5]. However, an isolated LBBB is occasionally found in individuals without clinically-detectable cardiovascular disease (CVD). While LBBB in older individuals and those with underlying heart disease is associated with increased mortality, it appears to have minimal effects on outcomes in younger, apparently healthy subjects [6–8]. Despite the latter, imaging studies have suggested left ventricular (LV) functional abnormalities in patients with isolated LBBB [9–12].

Prior two-dimensional (2D) (non-volumetric) transthoracic echocardiographic studies have shown a reduction in LV ejection fraction (LVEF) [9, 12] as well as an increase in LV cavity volume and mass in isolated LBBB [13, 14] but there are a paucity of data on cardiac volumes, mass and function in isolated LBBB using current gold-standard volumetric cardiovascular magnetic resonance (CMR) imaging. In the present study, we sought to characterize LV and right ventricular (RV) volumes and global systolic function, LV mass, and atrial volumes in adult subjects with an isolated LBBB and to determine whether those parameters differed from corresponding measurements among similar adults without LBBB. We compared patients with isolated LBBB but no clinically apparent CVD to age, sex, and body-size matched healthy controls, as well as against population-derived normal reference values via normalized Z-scores.

## Methods

### Selection of cases

We identified potential cases from two databases: the clinical echocardiography laboratory database at Beth Israel Deaconess Medical Center (BIDMC), and the Framingham Heart Study’s database of Offspring cohort members who underwent CMR during 2002–2006. The BIDMC database was queried to identify all patients referred for a resting transthoracic echocardiogram for assessment of a LBBB from January 2010 through December 2014. Among these patients, echocardiographic reports and other electronic medical record sources were reviewed. Any patient with an echocardiographic abnormality other than presence of abnormal septal motion typical of LBBB or greater than mild valvular disease, was excluded. Additionally, patients with any cardiac symptoms (angina, dyspnea/heart failure, palpitations, pre-syncope or syncope), known cardiovascular disease (coronary artery disease, cardiomyopathy, or arrhythmia), age > 85 years, diabetes, peripheral vascular disease, prior cerebrovascular events, or history of potential cardiotoxic chemotherapy exposure were excluded. This process identified 10 adults, who were invited to participate in the present study. Written informed consent was provided by each participant and the study was approved by the BIDMC Committee on Clinical Investigations; the study is in compliance with the Declaration of Helsinki. Verbal confirmation of the absence of CVD, diabetes, and use of chemotherapy were obtained from each subject. Each subject underwent an electrocardiogram (ECG) immediately prior to the CMR to confirm the presence of a LBBB on that day. We further identified 8 adults with isolated LBBB from among the 1794 members of the Framingham Offspring cohort who previously underwent CMR at BIDMC as part of a separate research study [15]. That study, and use of data then obtained in the present study, was approved by the institutional review boards of the BIDMC and the Boston University Medical Center and complies with the Declaration of Helsinki. Each Framingham participant provided written informed consent. Offspring participants have been followed closely since 1971 and have undergone periodic physical examination and ECG, as well as echocardiography and CMR. These 8 Offspring were verified to be free of clinical CVD (as described above) based on review of Framingham Offspring data and all available clinical records. A cardiologist reviewed the ECG performed at the Framingham Offspring examination cycle adjacent to CMR scanning to verify presence of LBBB.

### Selection of controls

Age-, sex-, hypertension-status and body-surface area (BSA)-matched controls (*n* = 18) were selected from among the Framingham Offspring cohort members who previously underwent CMR and were free of clinical CVD and LBBB. Hypertension was defined as a systolic blood pressure ≥ 140 mmHg, diastolic blood pressure ≥ 90 mmHg, or use of antihypertensive medication.

### CMR scanning and analysis

Non-contrast CMR was performed with study participants supine in a 1.5-T whole body scanner (Philips Healthcare, Best, The Netherlands), with a commercial 5-element cardiac array receiver coil. Following localizing scans, 2D end-expiratory breath-hold, ECG-gated, balanced steady-state free precession sequence cine images were obtained in the LV short-axis orientation encompassing both ventricles from base to apex (repetition time = R-R interval, TR = 3.2 ms, TE = 1.6 ms, flip angle 60 degrees, field-of-view 400 mm, matrix size 208 × 256, slice thickness 10 mm, no interslice gap, temporal resolution 30-40 ms). One slice was acquired with each 10–15 s breath- hold. The same hardware and imaging sequence was used to scan all study participants, including both controls and cases, regardless of whether cases were identified from the BIDMC or Framingham databases.

LV endocardial borders were manually traced at end-diastole and end-systole. LV epicardial borders were also traced at end-diastole. For consistency in analysis, LV trabeculations and papillary muscles were considered LV cavitary volume. Stroke volume (SV) was the difference between LV end-diastolic and end-systolic volumes. LVEF and RVEF were computed as SV divided by end-diastolic volume (EDV) in each ventricle. LV mass was calculated by multiplying the end-diastolic myocardial volume by myocardial density (1.05 g/ml) and indexed to BSA. Myocardial contraction fraction (MCF), a volumetric measure of myocardial shortening, was calculated as the ratio of LV SV to LV myocardial volume. Standard 2 chamber and 4 chamber cine images were obtained to determine left atrial (LA) and right atrial (RA) volumes by biplane method (LA volume (LAV) = 0.85×A_1_×A_2_ /L, where A_1_ and A_2_ were areas measured in 2 chamber and 4 chamber views, respectively, and L was the longest atrial length, and RA volume (RAV) = 0.85 × A^2^/L, where A was area measured in the 4 chamber view.

### Statistical analysis

Results from normally-distributed continuous data are expressed as mean ± standard deviation (SD). Non-indexed LV and RV parameters were compared between the isolated-LBBB group and matched controls using the Wilcoxon paired signed rank test; a *p* < 0.05 was considered significant. To compare ventricular parameters among isolated-LBBB patients with population-derived reference values, BSA-indexed (i) LV mass and biventricular volumes were converted to Z-scores (also known as standard scores) based on published sex and 10-year-age-group specific normal values. LV reference values were derived from 852 healthy adults free of any history of hypertension or CVD [15] and RV values from 1336 adults free of cardiopulmonary disease [16]. Specifically, each z-score was calculated as Z = (x-μ)/σ, where x is the individual measurement, and μ and σ respectively the corresponding mean and standard deviation for the appropriate sex and 10-year age group. Thus Z = + 1.5 would indicate that x was 1.5 standard deviations above the mean, whereas a Z = − 0.5 would indicate that x was half a standard deviation below the mean. Finally, we sought to determine whether LV or RV structural or global functional characteristics were associated with the degree of asynchrony, as assessed by the duration of the ECG QRS complex. Pearson correlation was used to assess possible linear relationships between biventricular z-scores for chamber size, ejection fraction, and cardiac index versus QRS duration.

## Results

Baseline characteristics are shown in Table 1. All subjects completed CMR imaging without complication and had interpretable images. The 18 adults with isolated LBBB ranged in age from 37 to 82 years and included 10 women. As expected, age, BSA, hypertension status and resting heart rate were similar (p = NS) between LBBB subjects and their matched controls.

**Table 1:** Baseline characteristics of subjects with left bundle branch block (LBBB) and controls

	LBBB (*N* = 18)	Controls (*N* = 18)	*P* Value
Age (years)	61.3 ± 13.0	61.8 ± 12.0	0.23
Female Sex (%)	55.6%	55.6%	–
BSA (m^2^)	1.90 ± 0.24	1.89 ± 0.23	0.42
Heart Rate (beats/min)	67 ± 11	65 ± 12	0.59

*BSA* body surface area, *LBBB* left bundle branch block

### Isolated LBBB vs. matched controls

**Fig. 1:**
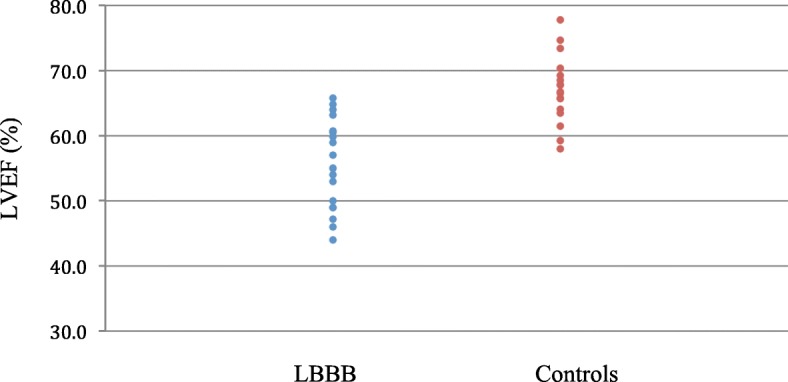
Individual LVEF data for subjects with and without an isolated LBBB. For the groups, mean values were 56 ± 7% vs. 68 ± 6%, *p* < 0.001

LV EDV, end-diastolic volume index (EDVi), end-systolic volume (ESV), and end-systolic volume index (ESVi) were greater in the LBBB group than among controls (all *p* < 0.02), but LV stroke volume and cardiac output were similar (both p = NS). The isolated LBBB group also had a lower LVEF (56 ± 7% vs. 68 ± 6%; *p* < 0.001, Fig. 1). Except for septal dyssynchrony, visually-assessed regional LV wall motion was normal in all LBBB subjects. There was no difference in LV mass, or any RV parameter, between LBBB subjects and controls (all p = NS; Table 2).

**Table 2:** Left and right ventricular parameters in isolated LBBB versus controls

	LBBB (*N* = 18)	Controls (*N* = 18)	*P* Value
LVEDV (ml)	145 ± 34	127 ± 28	0.01
LVEDVi (ml/m^2^)	76 ± 14	67 ± 10	0.014
LVEDD (mm)	53 ± 7	50 ± 5	0.07
LVESV (ml)	65 ± 20	42 ± 14	< 0.001
LVESVi (ml/m^2^)	34 ± 9	22 ± 6	< 0.001
LVSV (ml)	81 ± 18	86 ± 16	0.27
LVEF (%)	56 ± 7	68 ± 6	< 0.001
LV C.O. (L/min)	5.4 ± 1.4	5.3 ± 1.7	0.81
LV mass (g)	100 ± 25	105 ± 30	0.60
MCF	0.87 ± 0.20	0.89 ± 0.13	0.74
RVEDV (ml)	122 ± 38	130 ± 40	0.25
RVESV (ml)	46 ± 22	50 ± 21	0.35
RVSV (ml)	76 ± 21	80 ± 21	0.44
RVEF (%)	64 ± 9	63 ± 6	0.59
LAV (ml)	63 ± 20	75 ± 27	0.11
LAVi (ml/m^2^)	33 ± 8	39 ± 12	0.11
RAV (ml)	58 ± 30	56 ± 18	0.86
RAVi (ml/m^2^)	30 ± 8	29 ± 8	0.85

*LVEDV* left ventricular end-diastolic volume, *LVEDVi* left ventricular end-diastolic volume index, *LVESV* left ventricular end-systolic volume, *LVESVi* left ventricular end-systolic volume index, *LVSV* left ventricular systolic volume, *LVEF* left ventricular ejection fraction, *LV CO* left ventricular cardiac output, *LVM* left ventricular mass, *RVEDV* right ventricular end-diastolic volume, *RVESV* right ventricular end-systolic volume, *RVSV* right ventricular systolic volume, *RVEF* right ventricular ejection fraction, *LAV* left atrial volume, *LAVi* left atrial volume index, *MCF* myocardial contraction fraction, *RAV* right atrial volume, *RAVi* right atrial volume index, *LVEDD* left ventricular end-diastolic dimension

### Isolated LBBB vs. reference values

Z-scores were calculated based on sex and 10-year age group specific normal reference values derived from healthy members of the Framingham Offspring cohort. As expected, all mean Z-scores for the control group were within one-quarter standard deviation of the population average, apart from RVEF which was one-half standard deviation lower. In contrast, the isolated-LBBB group had LVEDVi 1.13 standard deviations greater than the general population, and an LVESVi of 2.33 greater. The combination of increased LVEDVi and substantially increased LVESVi resulted in markedly lower LVEF, with a Z = − 1.94, suggesting that on average, members of the isolated-LBBB group have lower LVEF than approximately 97% of the general population. Indexed RV volumes were not appreciably different from normal in either the isolated-LBBB or control groups. There was no significant difference in any atrial parameters (Tables 2 and 3).

**Table 3:** Aggregate Z-scores for isolated LBBB and control groups

	LBBB (*N* = 18)	Controls (*N* = 18)
LVEDVi (ml/m^2^)	1.13	0.09
LVEDD (mm)	0.44	−0.38
LVESVi (ml/m^2^)	2.33	−0.02
LVSVi (ml/m^2^)	− 0.22	0.22
LVEF (%)	-1.94	0.15
LV C.I. (L/min/m^2^)	0.10	−0.03
LVMi (g/m^2^)	−0.01	0.12
RVEDVi (ml/m^2^)	−0.09	0.12
RVESVi (ml/m^2^)	−0.01	0.19
RVSVi (ml/m^2^)	−0.17	−0.01
RVEF (%)	−0.31	−0.51
LAV (ml)	−0.27	0.29
LAVi (ml/m^2^)	−0.44	0.15
RAV (ml)	0.06	−0.04
RAVi (ml/m^2^)	−0.07	−0.15

### Electrocardiographic QRS duration and ventricular characteristics

Among the patients with isolated LBBB, QRS duration ranged from 126 to 158 ms (mean 142 ± 11 ms). Normalized LVEF (z-score) was inversely correlated with QRS duration with *r* = − 0.58 (*p* = 0.01), as was raw LVEF, *r* = − 0.56, *p* = 0.017. Both cardiac index and normalized cardiac index were inversely correlated with QRS duration; this was borderline significant for normalized cardiac index (*r* = − 0.48, *p* = 0.048) but not cardiac index (*r* = − 0.43, *p* = 0.077). LV volumes were not significantly correlated with QRS duration, nor were any RV parameters.

## Discussion

In this CMR study of healthy adults with an isolated LBBB, we found increased LV volumes and lower LVEF than age, sex, hypertension and BSA-matched individuals without LBBB. Compared to population means, LVEF among patients with isolated LBBB was nearly two standard deviations below that of healthy adults; this difference was principally attributable to increased LVESVi, which was over 2 SD greater than the population average. However, LV stroke volume, cardiac output and mass were similar to matched healthy controls and to population averages. Similarly, RV volumes and RVEF did not differ from matched controls or from population averages.

Prior investigators have used non-invasive cardiac imaging to examine LV volumes and LVEF in isolated LBBB. Radionuclide ventriculography was performed by Grines et al. in a study of 18 subjects with isolated LBBB to determine whether the abnormal septal motion in LBBB patients contributed to abnormalities in LV performance [9]. They found that apical and lateral regional ejection fractions were similar in LBBB patients and normal subjects. However, interventricular septal contribution to LVEF was strikingly diminished in LBBB compared with normal subjects (40 ± 16% versus 67 ± 7%, *p* < 0.001). As a result of abnormal septal contribution, global LVEF was reduced in LBBB patients (54 ± 7% vs 62 ± 15%). LV and RV volumes were not reported in their study. Similarly, in another study assessing intraventricular asynchrony by transthoracic echocardiography, Melek and colleagues also found a depressed biplane LVEF in isolated LBBB (54 ± 7% vs 61 ± 6%) [17]. However, despite finding a larger LVESV in LBBB group, and in contrast to our study, they found the LVEDV to be similar.

Data suggest that volumetric methods are superior to biplane methods for assessment of LV volumes [18]. van Dijk and colleagues used volumetric 3D transthoracic echocardiography and also found a reduction in LVEF associated with LBBB in asymptomatic patients (50 ± 9% vs 54 ± 5%), as well as an increase in LVEDV and LVESV (103 ± 37 ml vs 76 ± 27 ml and 52 ± 21 ml vs 36 ± 14 ml, respectively) [11, 19]. A volumetric CMR study by Valenti et al. also found a reduced LVEF (49 ± 7% vs 63 ± 5%) and an increased LVEDV index (91 ± 20 ml/m^2^ vs 75 ± 11 ml/m2) and LVESV index (47 ± 15 ml/m^2^ vs 28 ± 6 ml/m^2^) [20]. They also found a larger LV mass index in their LBBB group (63 ± 16 g/m^2^ vs 53 ± 12 g/m^2^, *p* = 0.04). In contrast to this and the 2D echocardiographic study of Vernooy et al. [14], we found no difference in LV mass associated with an isolated LBBB. The discordance between our results and the echocardiographic results may be due to the inaccuracy inherent to geometric assumptions and extrapolation of wall thickness with 2D echocardiography-derived LV mass.

Few data are available on RV volume and EF in isolated LBBB. Van Dijk et al. used 2D Doppler transthoracic echocardiography to assess RV dimensions and function in 15 patients with “asymptomatic” LBBB [21]. RV dimensions were assessed using the RV long axis measurement, RV tricuspid annulus diameter, and the RV area. RV function was assessed by RV fractional area change, M-mode determined tricuspid annular plane systolic excursion (TAPSE), and peak systolic velocity of the RV lateral wall annulus by tissue Doppler imaging. The asymptomatic LBBB cohort and the healthy subject cohort had similar RV dimensions and function.

The mechanism of the observed depressed LVEF in patients with an isolated LBBB is likely secondary to the altered septal electrical activation [9] leading to a delay in LV septal contraction compared with the RV. This abnormal septal motion results in an altered regional EF, with a diminished interventricular septal contribution to the global LV performance and LVEF. In addition, impairment of early diastolic blood flow in the left anterior descending coronary artery in patients with LBBB has also been postulated to be a potential cause for abnormal cardiac function [22, 23]. The mechanism of LV dilatation in LBBB has also been thought to be related to asynchronous electrical activation. In canine hearts, chronic pacing at the LV lateral wall has been shown to lead to LV dilatation [13]. Vernooy et al. demonstrated that the asynchronous ventricular activation during LBBB leads to redistribution of circumferential shortening and myocardial blood flow, and in the long run, leads to LV remodeling and dilatation. In the animal model of isolated LBBB, 8 weeks of biventricular stimulation reversed the functional and structural LV abnormalities [24], an observation concordant with controlled trials, where the decreases in echocardiographic LV volumes and increase in LVEF were significantly greater in cardiac resynchronization therapy recipients with than without LBBB [25, 26].

MCF is an independent measure of assessing myocardial shortening shown by King et al. to be useful for assessing differences in myocardial performance in patients with similar degree of hypertrophy [27]. In our study, in absence of centerline analysis, we used MCF, as a complementary method to assess global myocardial function and found no difference between MCF in the isolated LBBB cohort and the healthy subject cohort. This supports electromechanical dissociation, rather than intrinsic myocardial abnormality, as the explanation for the lower LVEF found in subjects with an isolated LBBB. Additionally, we found that greater electrocardiographic QRS duration was associated with lower LVEF, but not RVEF, further supporting the hypothesis that electrical, rather than intrinsic myocardial, dysfunction is primarily responsible for lower LVEF in the presence of isolated LBBB.

Our data are overall consistent with prior assessments of isolated LBBB, but augments the literature in several ways. We compared biventricular volumes and global systolic function among persons with isolated LBBB to age/gender matched controls as well as to population-based normative reference values. Use of the Z-score allowed us to quantify deviation from normal in terms of easily interpreted standard deviations. Additionally, we present biventricular results in the same subjects, using image data acquired in the same scanning session. Finally, we used volumetric CMR, which is widely considered the gold standard for determination of ventricular volumes and EF.

Our study has several limitations. Similar to prior studies on this topic, the isolated LBBB cohort is relatively small, despite screening four consecutive years of patients from the BIDMC clinical echocardiography database and a subset of the Framingham Offspring cohort. While this is likely a result of the relative rarity of isolated LBBB, our methods allowed us to exclude CVD with high confidence based on review of hospital electronic medical records and extensive Framingham data. CMR contrast was not used and thus no late gadolinium enhancement images were available to assess for occult coronary artery disease or focal fibrosis, but no subject had cine CMR evidence for regional dysfunction other than the septum. Furthermore, our control population had not undergone LGE evaluation and therefore comparison between the two groups would not have been possible. We did not study differences between ECG patterns of LBBB often seen in CAD or myopathic patients, as our subjects were free of these disorders. Finally, data regarding the duration of LBBB and its impact on long-term outcomes are unknown.

## Conclusions

Using the reference standard of volumetric CMR, adults with an isolated LBBB have greater LV volumes, lower global LVEF, and similar LV mass as compared with age, sex, hypertension-status, and BSA-matched individuals. RV size and function as well as atrial anatomy are similar. These data are important to consider in the clinical interpretation of LV and RV volumes and EF, LV mass, and atrial anatomy in isolated LBBB patients referred for CMR.

## Abbreviations

2D: Two-dimensional
BIDMC: Beth Israel Deaconess Medical Center
BSA: Body-surface area
CMR: Cardiovascular magnetic resonance
CVD: Cardiovascular disease
ECG: Electrocardiogram
EDV: End-diastolic volume
EDVi: End-diastolic volume index
EF: Ejection fraction
ESV: End-systolic volume
ESVi: End-systolic volume index
LA: Left atrium/left atrial
LAV: Left atrial volume
LAVi: Left atrial volume index
LBBB: Left bundle-branch block
LV: Left ventricular/left ventricle
LVEDD: Left ventricular end diastolic dimension
LVEF: Left ventricular ejection fraction
MCF: Myocardial contraction fraction
RA: Right atrium/right atrial
RAV: Right atrial volume
RAVi: Right atrial volume index
RV: Right ventricular/right ventricle
RVEF: Right ventricular ejection fraction
SD: Standard deviation
SV: Stroke volume

## References

[1] Hiss RG, Lamb LE (1962). Electrocardiographic findings in 122,043 individuals. Circulation.

[2] Ostrander LD, Brandt RL, Kjelsberg MO, Epstein FH (1965). Electrocardiographic findings among the adult population of a Total natural community, Tecumseh, Michigan. Circulation.

[3] Siegman-Igra Y, Yahini JH, Goldbourt U, Neufeld HN (1978). Intraventricular conduction disturbances: a review of prevalence, etiology, and progression for ten years within a stable population of Israeli adult males. Am Heart J.

[4] Eriksson P, Hansson PO, Eriksson H, Dellborg M (1998). Bundle-branch block in a general male population: the study of men born 1913. Circulation.

[5] Francia P, Balla C, Paneni F, Volpe M (2007). Left bundle-branch block--pathophysiology, prognosis, and clinical management. Clin Cardiol.

[6] Rotman M, Triebwasser JH (1975). A clinical and follow-up study of right and left bundle branch block. Circulation.

[7] Zhang ZM, Rautaharju PM, Soliman EZ (2012). Mortality risk associated with bundle branch blocks and related repolarization abnormalities (from the Women's Health Initiative [WHI]). Am J Cardiol.

[8] Eriksson P, Wilhelmsen L, Rosengren A (2005). Bundle-branch block in middle-aged men: risk of complications and death over 28 years. The primary prevention study in Goteborg, Sweden. Eur Heart J.

[9] Grines CL, Bashore TM, Boudoulas H, Olson S, Shafer P, Wooley CF (1989). Functional abnormalities in isolated left bundle branch block. The effect of interventricular asynchrony. Circulation.

[10] Duzenli MA, Ozdemir K, Soylu A, Aygul N, Yazici M, Tokac M (2008). The effect of isolated left bundle branch block on the myocardial velocities and myocardial performance index. Echocardiography.

[11] van Dijk J, Dijkmans PA, Gotte MJ, Spreeuwenberg MD, Visser CA, Kamp O (2008). Evaluation of global left ventricular function and mechanical dyssynchrony in patients with an asymptomatic left bundle branch block: a real-time 3D echocardiography study. Eur J Echocardiogr.

[12] Ozdemir K, Altunkeser BB, Danis G (2001). Effect of the isolated left bundle branch block on systolic and diastolic functions of left ventricle. J Am Soc Echocardiogr.

[13] van Oosterhout MF, Prinzen FW, Arts T (1998). Asynchronous electrical activation induces asymmetrical hypertrophy of the left ventricular wall. Circulation.

[14] Vernooy K, Verbeek XA, Peschar M (2005). Left bundle branch block induces ventricular remodelling and functional septal hypoperfusion. Eur Heart J.

[15] Yeon SB, Salton CJ, Gona P (2015). Impact of age, sex, and indexation method on MR left ventricular reference values in the Framingham heart study offspring cohort. J Magn Reson Imaging.

[16] Foppa M, Arora G, Gona P (2016). Right ventricular volumes and systolic function by cardiac magnetic resonance and the impact of sex, age, and obesity in a longitudinally followed cohort free of pulmonary and cardiovascular disease: the Framingham heart study. Circ Cardiovasc Imaging.

[17] Melek M, Esen O, Esen AM, Barutcu I, Onrat E, Kaya D (2006). Tissue Doppler evaluation of intraventricular asynchrony in isolated left bundle branch block. Echocardiography.

[18] Chuang ML, Hibberd MG, Salton CJ (2000). Importance of imaging method over imaging modality in noninvasive determination of left ventricular volumes and ejection fraction: assessment by two- and three-dimensional echocardiography and magnetic resonance imaging. J Am Coll Cardiol.

[19] Brunekreeft JA, Graauw M, de Milliano PA, Keijer JT (2007). Influence of left bundle branch block on left ventricular volumes, ejection fraction and regional wall motion. Neth Heart J.

[20] Valenti V, Zia MI, Shubayev L (2012). Cardiac magnetic resonance evaluation of the impact of interventricular and intraventricular dyssynchrony on cardiac ventricular systolic and diastolic function in patients with isolated left bundle branch block. Am J Cardiol.

[21] van Dijk J, Knaapen P, Bekkering I, Gotte MJ, Kamp O (2008). Right ventricular dimensions and function in isolated left bundle branch block: is there evidence of biventricular involvement?. Echocardiography.

[22] Youn HJ, Park CS, Cho EJ (2005). Left bundle branch block disturbs left anterior descending coronary artery flow: study using transthoracic Doppler echocardiography. J Am Soc Echocardiogr.

[23] Skalidis EI, Kochiadakis GE, Koukouraki SI, Parthenakis FI, Karkavitsas NS, Vardas PE (1999). Phasic coronary flow pattern and flow reserve in patients with left bundle branch block and normal coronary arteries. J Am Coll Cardiol.

[24] Vernooy K, Cornelussen RN, Verbeek XA (2007). Cardiac resynchronization therapy cures dyssynchronopathy in canine left bundle-branch block hearts. Eur Heart J.

[25] Gold MR, Thebault C, Linde C (2012). Effect of QRS duration and morphology on cardiac resynchronization therapy outcomes in mild heart failure: results from the resynchronization reverses remodeling in systolic left ventricular dysfunction (REVERSE) study. Circulation.

[26] Hsu JC, Solomon SD, Bourgoun M (2012). Predictors of super-response to cardiac resynchronization therapy and associated improvement in clinical outcome: the MADIT-CRT (multicenter automatic defibrillator implantation trial with cardiac resynchronization therapy) study. J Am Coll Cardiol.

[27] King Donald L, El-Khoury Coffin Lyna, Maurer Mathew S (2002). Myocardial contraction fraction: a volumetric index of myocardial shortening by freehand three-dimensional echocardiography. Journal of the American College of Cardiology.

